# Supplementary material for: Patient‐specific logic models of signaling pathways from screenings on cancer biopsies to prioritize personalized combination therapies
Source: Mol Syst Biol. 2020 Feb 19;16(2):e8664. doi: 10.15252/msb.20188664 (PMC7029724; doi:10.15252/msb.20188664)
Supplement: Supplementary file 1 — Appendix [file MSB-16-e8664-s001.pdf]

Appendix to: **Patient-specific logic models of signaling pathways from screenings on cancer biopsies to prioritize personalized combination therapies**

Federica Eduati[1,2,3,4], Patricia Jaaks[5], Jessica Wappler[6], Thorsten Cramer[6,7], Christoph A. Merten[1], Mathew J. Garnett[5], Julio Saez-Rodriguez[2,3,8,*]

[1] European Molecular Biology Laboratory (EMBL), Genome Biology Unit, Meyerhofstrasse 1, Heidelberg, Germany

[2] European Molecular Biology Laboratory, European Bioinformatics Institute (EMBL-EBI), Wellcome Trust Genome Campus, Hinxton, UK

[3] Joint Research Centre for Computational Biomedicine (JRC-COMBINE), RWTH Aachen University, Faculty of Medicine, Aachen, Germany

[4] Dept. Biomedical Engineering, Eindhoven University of Technology, Eindhoven, The Netherlands

[5] Wellcome Trust Sanger Institute, Wellcome Trust Genome Campus, Hinxton, Cambridgeshire, United Kingdom

[6] Molecular Tumor Biology, Department Surgery, RWTH University Hospital, 52057 Aachen, Germany

[7] ESCAM – European Surgery Center Aachen Maastricht, Aachen, Germany and Maastricht, The Netherlands.

[8] Institute for Computational Biomedicine, Heidelberg University, Faculty of Medicine, BIOQUANT-Center, 69120 Heidelberg, Germany

*corresponding author: julio.saez@bioquant.uni-heidelberg.de

Content:

Appendix Figure S1. Performances of models optimised for each cell line.
Appendix Figure S2. Sensitivity analysis on model parameters.
Appendix Figure S3. Sensitivity analysis on model predictions.
Appendix Figure S4. Differentially expressed genes.
Appendix Figure S5. Full list of cell line-specific model predictions.
Appendix Figure S6. Combination of MK-2206 (Akt inhibitor) and Trametinib (MEK inhibitor).
Appendix Figure S7. Combination of PHT-427(Akt and PDPK1 inhibitor) and Navitoclax (Bcl-2 family inhibitor).
Appendix Figure S8. Combination of Taselisib (PI3K inhibitor) and Navitoclax (Bcl-2 family inhibitor).
Appendix Figure S9. Clustering of patient based on data and parameter values.
Appendix Figure S10. Measurement error of the MPS data.

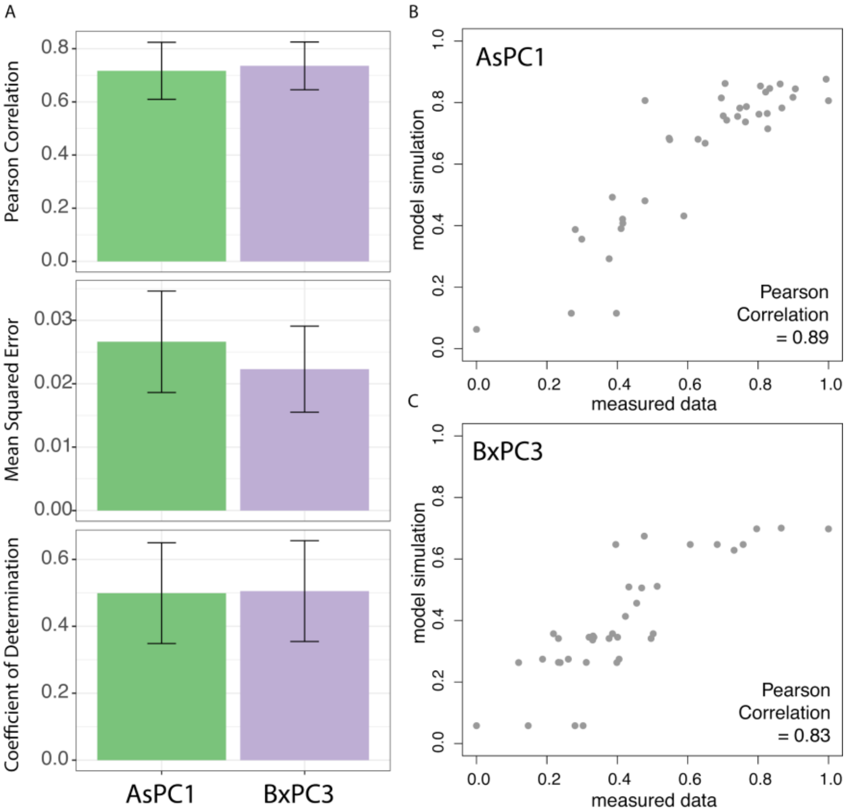

Appendix Figure S1. Performances of models optimised for each cell line.

(A) Scores of optimised model based on different metrics (Pearson correlation, Mean squared error and Coefficient of determination). Optimisation for each cell line was repeated 10 times and mean and standard deviation are reported for each metric. (B, C) Measured versus simulated data across all experimental conditions for the optimal models.

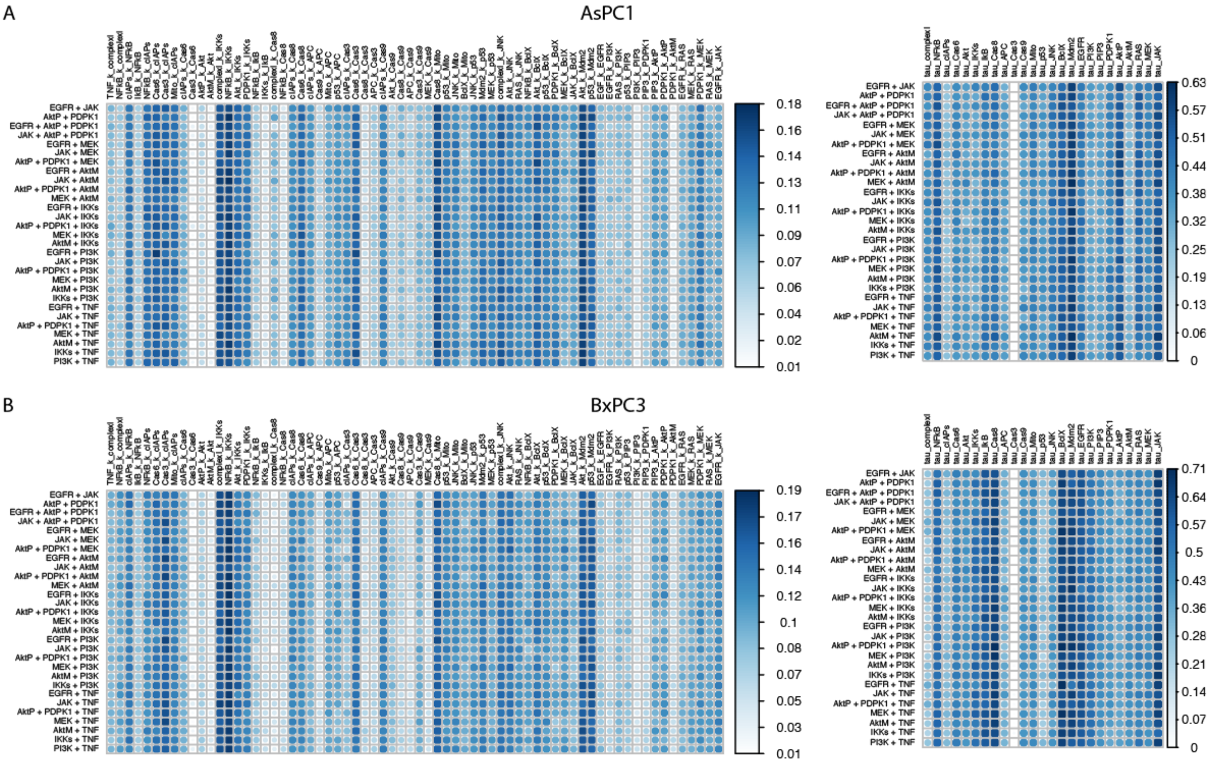

Appendix Figure S2. Sensitivity analysis on model parameters.

Variance of the estimated parameters (columns) across all the bootstrap iterations where a specific drug combination (rows) was in the validation set (i.e. left out from the training set because of the resampling with replacement).

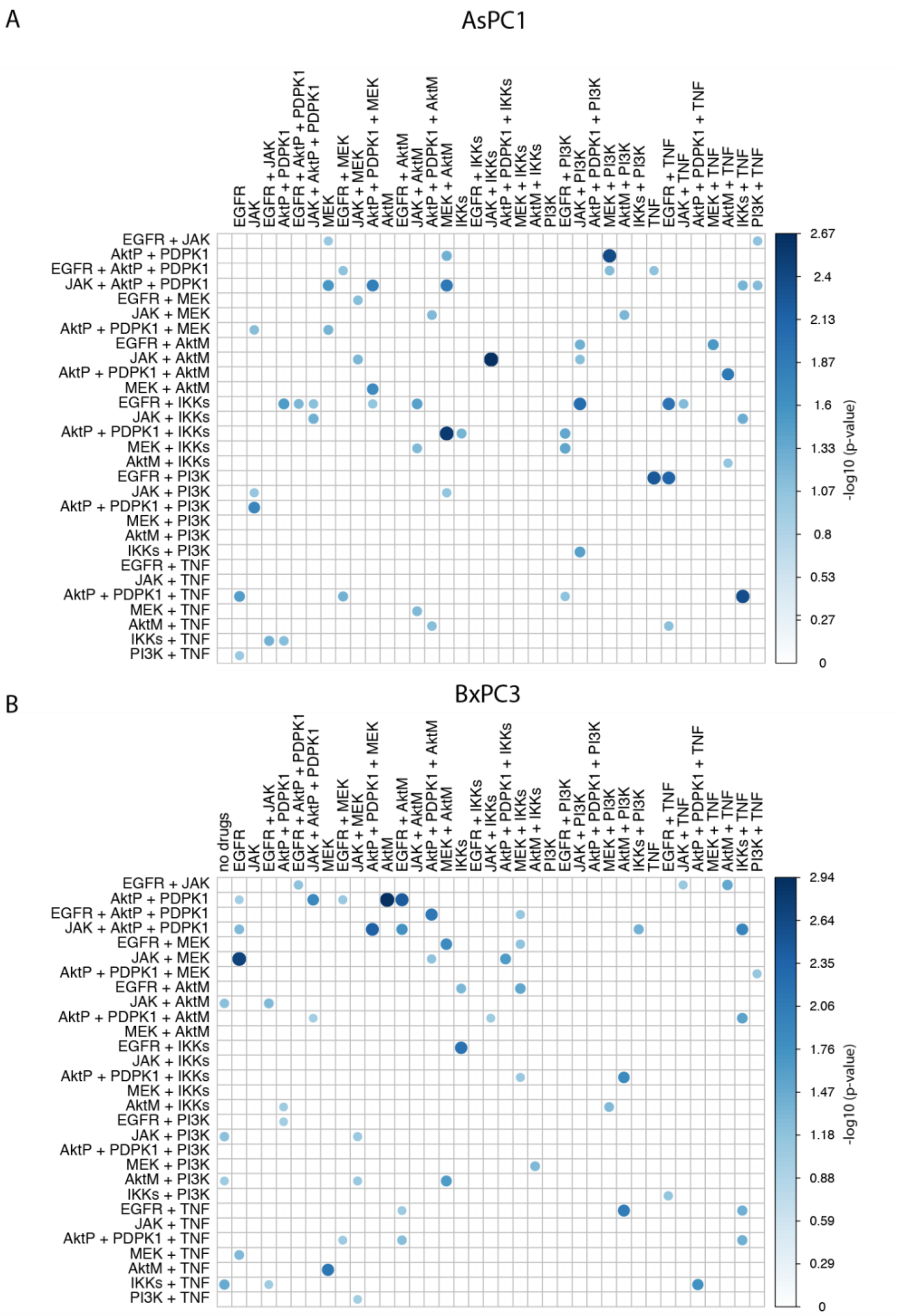

Appendix Figure S3. Sensitivity analysis on model predictions.

Analysis of the associations between the prediction error for a specific drug combination (rows) and all the experiments in the training set (columns). The analysis was performed by looking at the bootstrap iterations when the specific drug combination was in the validation set (i.e. left out from the training set because of the resampling with replacement) and performed linear regression considering as input variable a matrix with 0 or 1 (depending on whether a condition was in the training set or not), and as output vector the corresponding squared error of the prediction of the left-out condition. The heatmap shows the -log10 of the p-values only when the p-value is < 0.1.

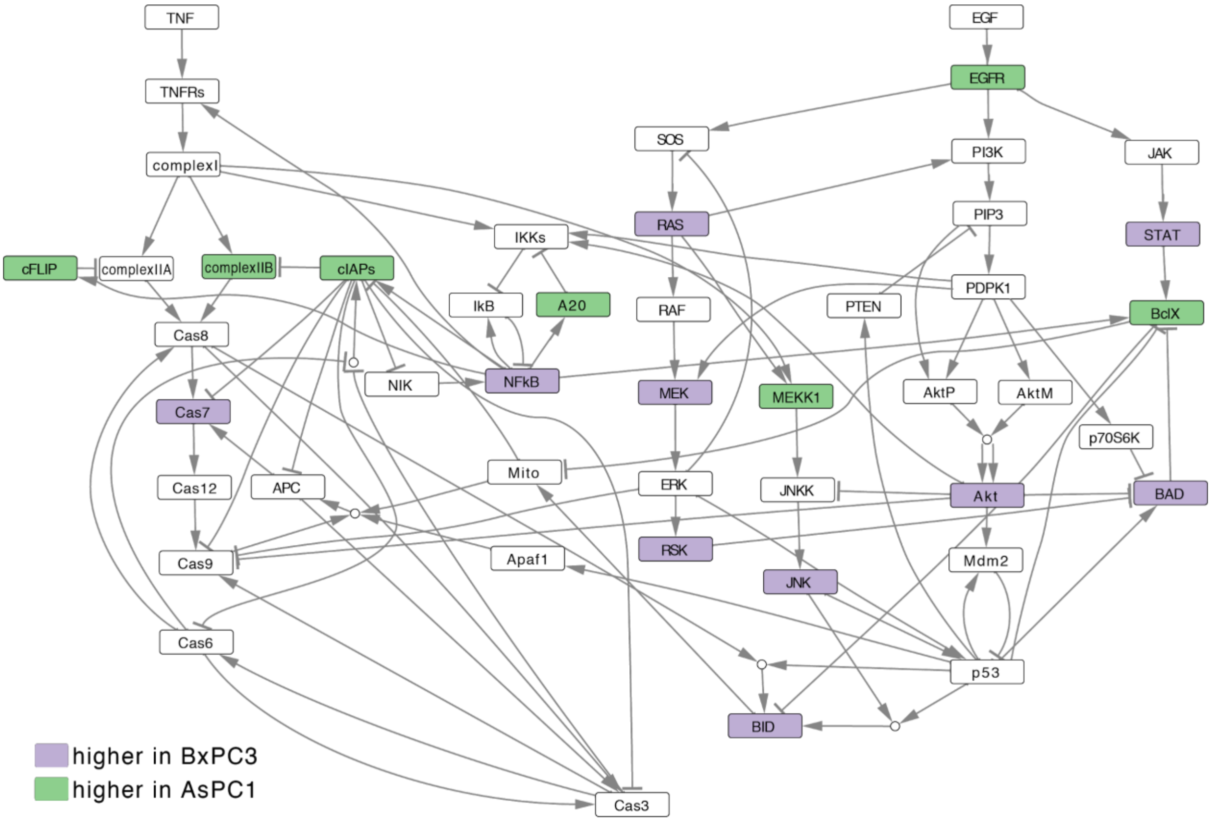

Appendix Figure S4. Differentially expressed genes.

Microarray data downloaded from GDSC database (https://www.cancerrxgene.org, Iorio et al., Cell, 2016) were used to check which genes in our pathways of interest are differentially expressed between the two cell lines (i.e. log2 fold change > 1).

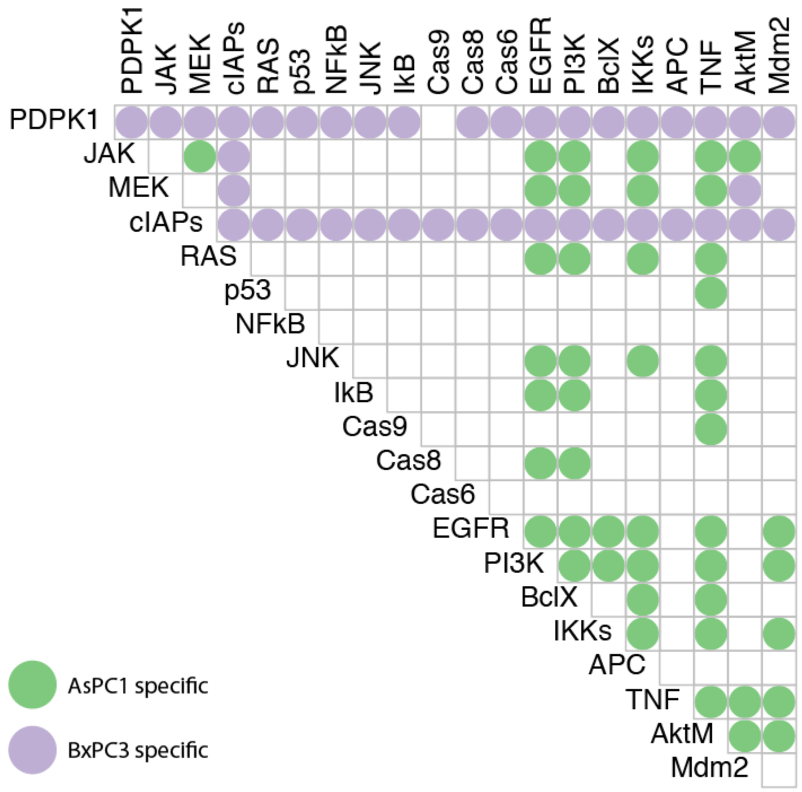

Appendix Figure S5. Full list of cell line-specific model predictions.

New drug combinations predicted to be highly specific for each cell line: higher predicted Cas3 in BxPC3 than in AsPC1 cells are shown in purple, while the ones significantly more specific for AsPC1 are shown in green.

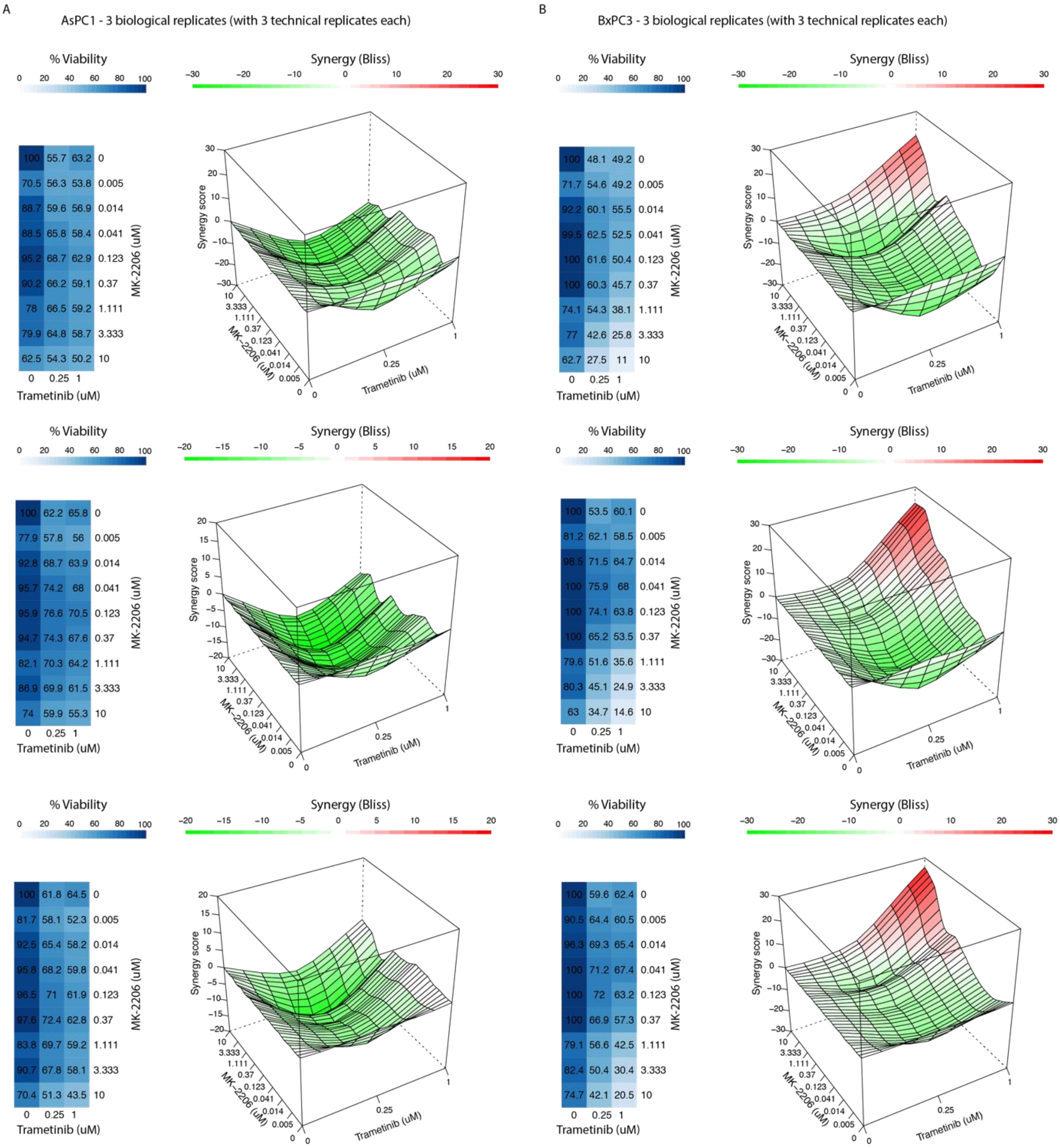

Appendix Figure S6. Combination of MK-2206 (Akt inhibitor) and Trametinib (MEK inhibitor). *All data and synergy scores (Bliss independence) are shown for the 3 biological replicates (top, central and lower panel) for (A) AsPC1 and (B) BxPC3. Viability data (% with respect to untreated control, averaged across technical replicates) are shown as heatmap. The 3D curves represent the synergy scores computed using the Bliss independence model from the 'synergyfinder' R package (He et al, 2018).*

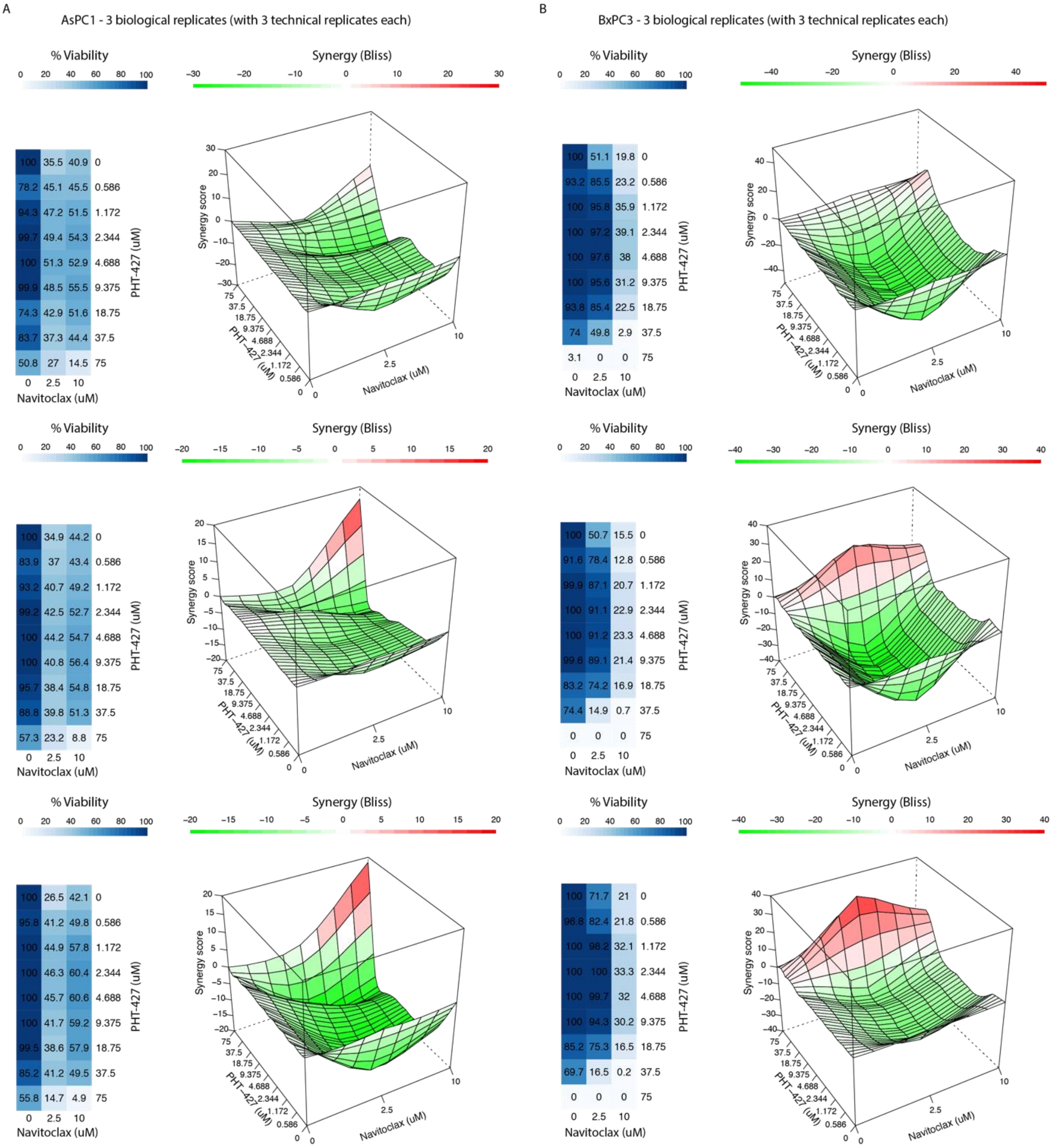

Appendix Figure S7. Combination of PHT-427(Akt and PDPK1 inhibitor) and Navitoclax (Bcl-2 family inhibitor).

All data and synergy scores (Bliss independence) are shown for the 3 biological replicates (top, central and lower panel) for (A) AsPC1 and (B) BxPC3. Viability data (% with respect to untreated control, averaged across technical replicates) are shown as heatmap. The 3D curves represent the synergy scores computed using the Bliss independence model from the 'synergyfinder' R package (He et al, 2018).

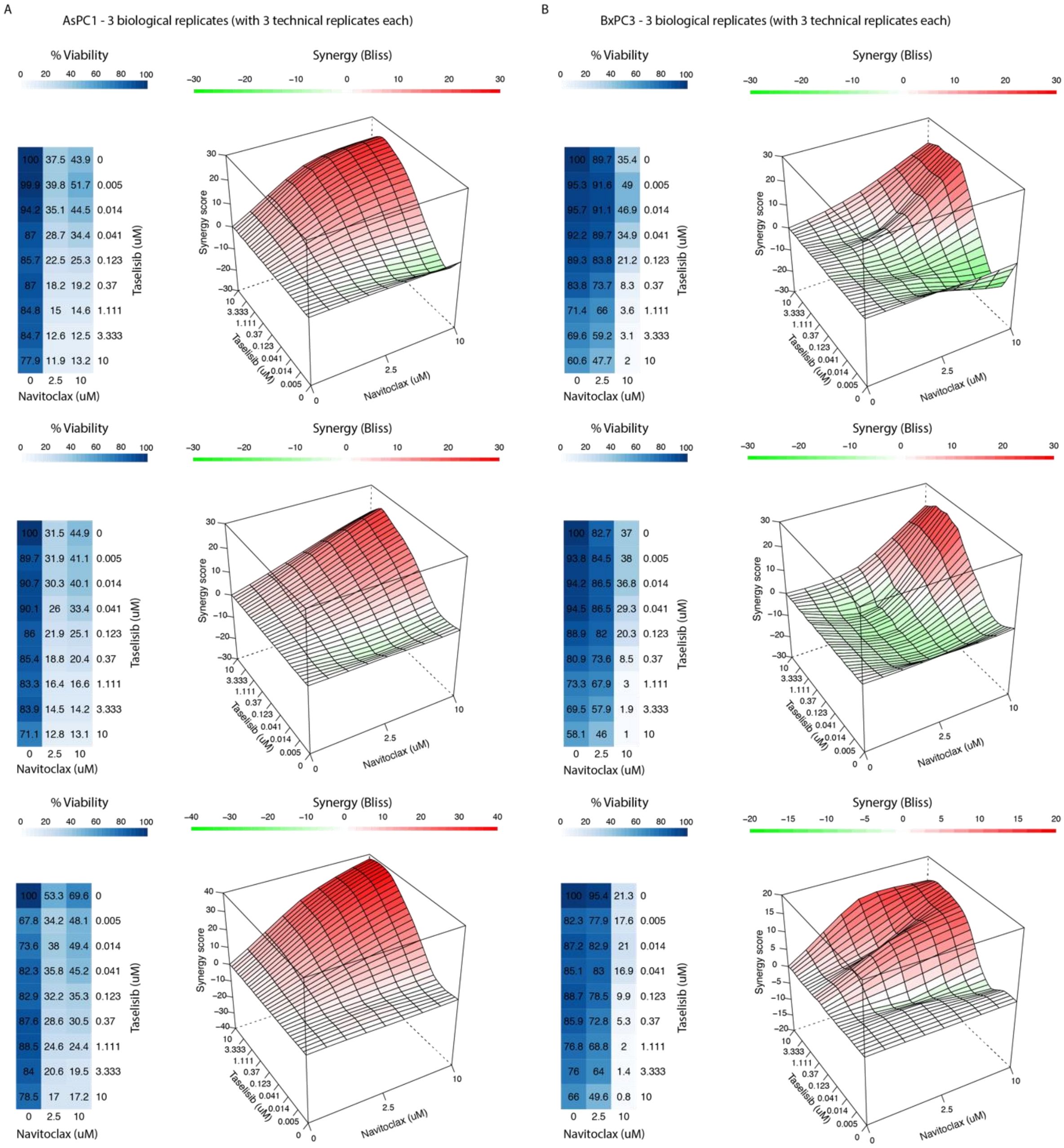

Appendix Figure S8. Combination of Taselisib (PI3K inhibitor) and Navitoclax (Bcl-2 family inhibitor).

All data and synergy scores (Bliss independence) are shown for the 3 biological replicates (top, central and lower panel) for (A) AsPC1 and (B) BxPC3. Viability data (% with respect to untreated control, averaged across technical replicates) are shown as heatmap. The 3D curves represent the synergy scores computed using the Bliss independence model from the 'synergyfinder' R package (He et al, 2018).

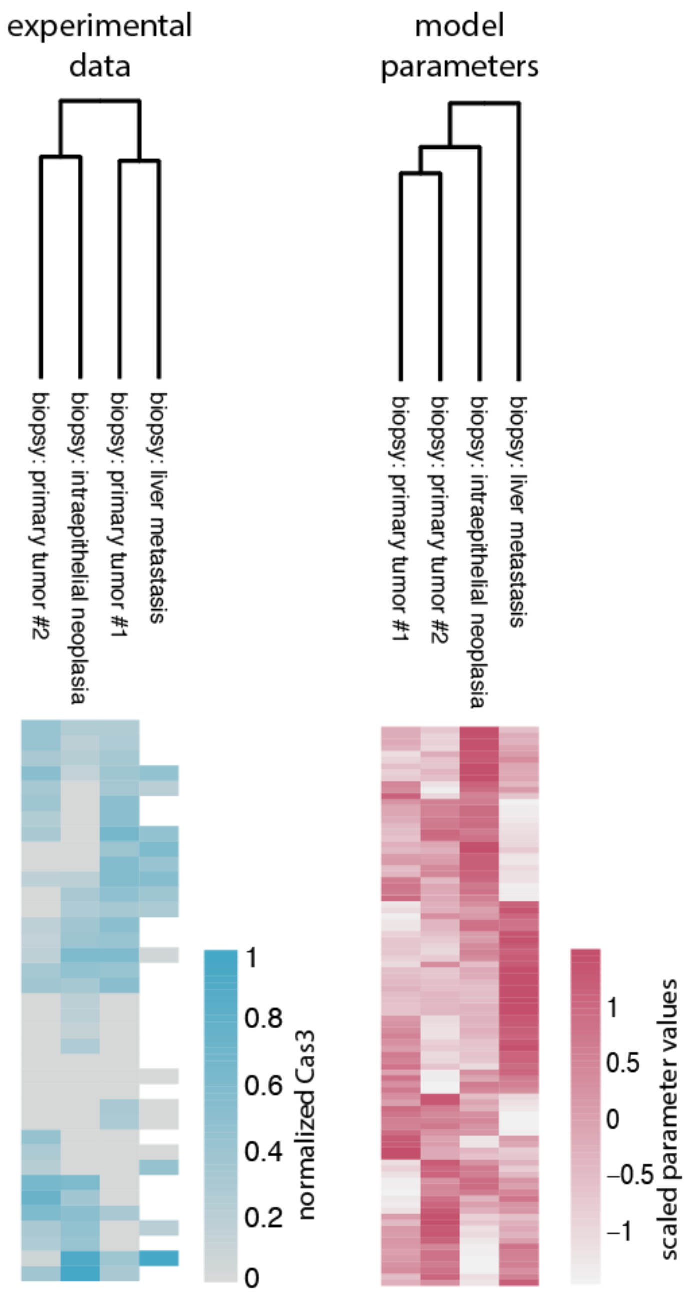

Appendix Figure S9. Clustering of patient based on data and parameter values.

Hierarchical clustering (Eucledian distance, complete-linkage) of experimental MPS data (left) and median estimated model parameters (right) for the four pancreatic cancer patients. Model parameter better recapitulate similarities between different tumor stages.

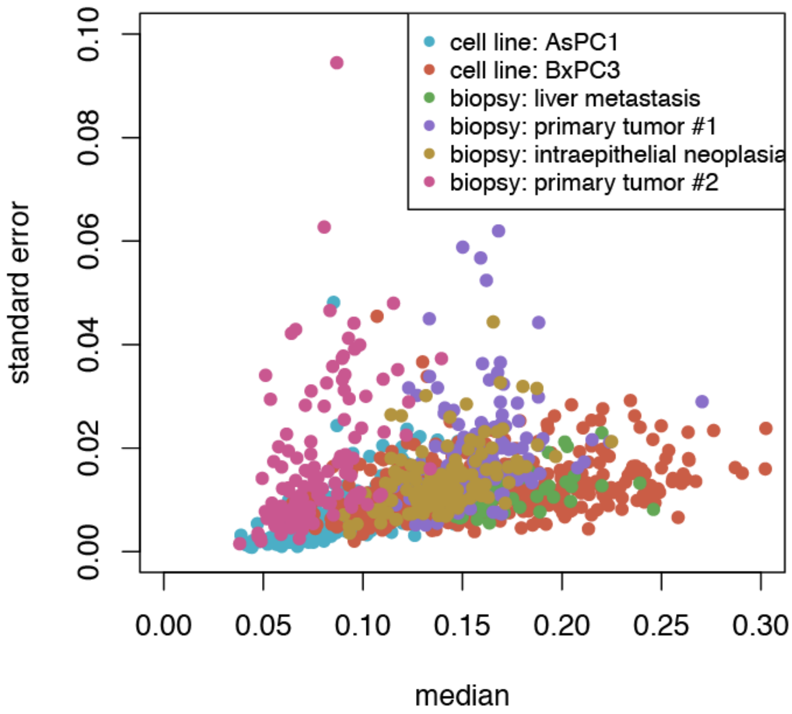

Appendix Figure S10. Measurement error of the MPS data.

Plot of median and standard error for the data from the Microfluidics Perturbation Screenings (MPS) for each individual sample (cell lines and patients) and each individual run, before computing z-score and normalizing.
